# Real-World Outcomes of Colorectal Signet Ring Cell Carcinoma: Clinicopathological Characteristics, Treatment Patterns, and Survival

**DOI:** 10.7759/cureus.114580

**Published:** 2026-08-15

**Authors:** Arul Kumar A, Vasireddy Sadasivudu, Harsh Karun, Abhishek Ganguly, Yalini Devi, Potireddy Yaswanth Reddy, Mayank Tripathi, Ankita Pal

**Affiliations:** 1 Surgical Oncology, Mahamana Pandit Madan Mohan Malaviya Cancer Centre and Homi Bhabha Cancer Hospital, Homi Bhabha National Institute, Varanasi, IND; 2 Surgical Oncology, Homi Bhabha National Institute, Mumbai, IND; 3 Biostatistics, Mahamana Pandit Madan Mohan Malaviya Cancer Centre and Homi Bhabha Cancer Hospital, Homi Bhabha National Institute, Varanasi, IND; 4 Biostatistics, Homi Bhabha National Institute, Mumbai, IND

**Keywords:** colorectal cancer, overall survival, rectal cancer, signet-ring cell carcinoma, treatment outcomes

## Abstract

Background: Colorectal signet-ring cell carcinoma (SRCC) is a rare and aggressive histological subtype of colorectal cancer (CRC) characterised by advanced stage at presentation and poor prognosis. Data from the Indian subcontinent remain limited. This study aimed to evaluate the clinicopathological characteristics, treatment patterns, prognostic factors, and survival outcomes of patients with colorectal SRCC treated at a tertiary cancer centre.

Methods: A retrospective observational study was conducted, including patients with histologically confirmed colorectal SRCC treated at a tertiary cancer centre. Demographic, clinical, pathological, treatment, and survival data were collected. Overall survival (OS) was estimated using the Kaplan-Meier method. Prognostic factors associated with OS were evaluated using univariate and multivariable Cox proportional hazards regression analyses.

Results: A total of 263 patients were included. The median age was 37 years (IQR, 28.5-49), with 58.2% of patients younger than 40 years, and males constituted 63.1% of the cohort. The rectum was the predominant primary site (73.4%), and 93.2% of patients presented with stage III or IV disease. Curative-intent treatment was performed in 73.8% of patients, with total neoadjuvant therapy followed by surgery being the most common treatment strategy (27.2%). Among patients receiving neoadjuvant treatment, a complete response was achieved in 11.0%, whereas disease progression occurred in 22.9%. Recurrence developed in 48.6% of surgically treated patients, with the peritoneum representing the most common site of recurrence (36.4%). After a median follow-up of 35 months, the median DFS was 21 months (95% CI: 15.8-26.1), with a two-year DFS rate of 40.3±4.5%, while the median OS was 30 months (95% CI: 26.1-33.9) with a two-year OS rate of 62.1±4.4%. On univariate analysis, positive lymph node status, lymphovascular invasion, perineural invasion, positive resection margins, and elevated baseline carcinoembryonic antigen (CEA) were significantly associated with worse OS, whereas neoadjuvant therapy was associated with improved survival. In multivariable analysis, pathological nodal stage, positive resection margins, and elevated baseline CEA showed trends towards poorer survival but did not reach statistical significance.

Conclusion: Colorectal SRCC predominantly affects young patients and presents at an advanced stage with aggressive clinicopathological features and poor oncological outcomes. Advanced nodal disease, adverse pathological features, and elevated baseline CEA were associated with inferior survival. Early diagnosis, multidisciplinary management, and prospective multicentre studies incorporating comprehensive molecular profiling are required to improve outcomes in this challenging disease.

## Introduction

Colorectal cancer (CRC) is the third most diagnosed cancer and the second leading cause of cancer-related mortality worldwide [1]. Signet-ring cell carcinomas (SRCCs) are an extremely aggressive yet uncommon variant of CRC. Worldwide, SRCC accounts for approximately 1% of all colorectal cancers. Although population-based data from India are limited, several institutional series from the Indian subcontinent have reported a relatively higher proportion of SRCC among patients with colorectal cancer, ranging from approximately 15% to 20% [2,3].

SRCC was first described in the colorectum by Laufman and Saphir in 1951 as “linitis plastica” of the colon [4]. It classically consists of cells with peripherally placed nuclei displaced by intracytoplasmic mucin in >50% of the total tumour mass. Signet-ring cells occur either as single cells or as loose clusters and spread diffusely throughout the bowel wall, implying loss of cell-cell adhesion at the molecular level [5].

Histological subtypes have emerged as an important prognostic determinant in colorectal cancer. Among the different histological variants, SRCC and mucinous carcinoma (MC) are characterised by a more aggressive clinical phenotype, frequently presenting at an advanced stage with a higher burden of locoregional and distant disease compared with conventional adenocarcinoma (AC) [6]. In addition to presenting at an advanced stage, SRCC has historically been associated with limited responsiveness to conventional treatment modalities and inferior survival outcomes compared with conventional AC [7]. Whether the unfavourable prognosis associated with SRCC and MC is solely attributable to delayed stage at diagnosis or reflects the intrinsic biological and molecular aggressiveness of these tumour subtypes remains incompletely understood.

In view of the limited data on colorectal SRCC, particularly from the Indian population, the primary objective of this study was to characterise the clinicopathological features and survival outcomes of patients with colorectal SRCC treated at a tertiary cancer centre. Secondary objectives were to describe treatment patterns and evaluate clinicopathological factors associated with overall survival.

## Materials and methods

Study design and patient selection

In this study, we retrospectively evaluated the data of patients diagnosed with colorectal AC with signet-ring cell differentiation at the Mahamana Pandit Madan Mohan Malviya Cancer Centre, Varanasi, from June 2018 to December 2025. The data were extracted from the hospital electronic medical records and pathology reports. All patients aged >10 years and <75 years with pure signet-ring cell AC of the colorectum were included. Tumours with associated mucinous features were included only if the signet-ring cell component constituted >50% of the tumour, in accordance with the WHO definition. Variables evaluated included patient characteristics, such as age, sex, presenting symptoms, nutritional status, and previous treatment history. Nutritional status was assessed retrospectively using the baseline body mass index (BMI) and serum albumin level documented in the medical records before initiation of treatment. Tumour characteristics evaluated included tumour site, colonoscopy findings, digital rectal examination (DRE) findings, carcinoembryonic antigen (CEA) levels, biopsy findings, and clinical staging, as well as treatment details, including neoadjuvant treatment, surgical management received, postoperative histopathological features, CEA, follow-up, recurrence, and survival data.

Contrast-enhanced computed tomography (CECT) of the thorax, abdomen, and pelvis was the routine staging investigation, whereas whole-body PET-CECT was performed in some cases. All patients were staged according to the American Joint Committee on Cancer (AJCC) Cancer Staging Manual (8th Edition) for colon or rectal cancer, as appropriate. MRI of the pelvis was performed for all patients with rectal cancer requiring local staging. Patients with locally advanced rectal cancer received neoadjuvant treatment following multidisciplinary tumour board discussion. Long-course chemoradiotherapy consisted of external beam radiotherapy to a total dose of 50 Gy in 25 fractions, with concurrent fluoropyrimidine-based chemotherapy (oral capecitabine 825 mg/m² twice daily on radiotherapy days or continuous infusion of 5-fluorouracil). Short-course radiotherapy, when indicated, consisted of 25 Gy in five fractions followed by delayed surgery according to institutional protocols. Neoadjuvant chemotherapy alone consisted primarily of CAPOX (capecitabine 1000 mg/m² twice daily on days 1-14 plus oxaliplatin 130 mg/m² on day 1, every three weeks) or mFOLFOX6. Total neoadjuvant therapy (TNT) incorporated systemic chemotherapy administered before and/or after chemoradiotherapy, followed by definitive surgery. The choice of neoadjuvant strategy was individualised based on tumour location, clinical stage, resectability, patient performance status, and recommendations from the multidisciplinary tumour board.

Response to neoadjuvant treatment was assessed using serial serum CEA levels and radiological imaging with contrast-enhanced computed tomography (CECT) of the thorax, abdomen, and pelvis and/or magnetic resonance imaging (MRI) of the pelvis, as appropriate. For patients with rectal cancer, treatment response was evaluated using the MRI Tumor Regression Grade (mrTRG) system. The overall response was categorised as complete response, partial response, stable disease, or progressive disease based on radiological assessment and multidisciplinary team review. The mode of surgery, open vs MIS, was decided on a case-by-case basis based on patient and tumour characteristics and extent of disease. All patients were discussed at the MDT to determine an optimal treatment plan.

Statistical analysis

Baseline demographic, clinical, pathological, and treatment-related variables were summarised using descriptive statistics. Continuous variables were presented as the median and interquartile range (IQR), whereas categorical variables were expressed as frequencies and percentages. Associations between categorical variables, particularly treatment modalities and post-treatment response categories, were evaluated using Fisher's exact test. Time-to-event outcomes included overall survival (OS), defined as the interval from the date of surgery to the date of death from any cause, and disease-free survival (DFS), defined as the interval from the date of curative surgery to the date of the first documented recurrence or death from any cause, whichever occurred first. These outcomes were estimated using the Kaplan-Meier method, with survival probabilities reported as median survival, mean survival with 95% confidence intervals (CIs), and two-year survival rates. Prognostic factors influencing OS and DFS were initially evaluated using univariate Cox proportional hazards regression analysis, and variables considered clinically relevant or statistically significant were subsequently entered into multivariable Cox proportional hazards models. Hazard ratios (HRs) with corresponding 95% CIs were reported. All statistical tests were two-sided, and a p-value <0.05 was considered statistically significant.

## Results

The cohort included 263 patients (Table 1), with a median age of 37 years (IQR: 28.5-49 years). More than half of the patients, 153 (58.2%), were younger than 40 years, while 110 (41.8%) were aged >40 years, suggesting that signet-ring cell/mucin-producing colorectal carcinoma predominantly affected younger individuals in this population. Males constituted nearly two-thirds of the study population, 166 (63.1%), resulting in a male-to-female ratio of approximately 1.7:1. Bleeding per rectum was the most common presenting symptom, reported in 154 (58.6%) patients. 

**Table 1:** Summarisation of demographic and clinical characteristics (n = 263) MSI: microsatellite instability, MSS: microsatellite stable, pMMR: mismatch repair proficient.

Characteristic	Value
Age, years	
Median (IQR)	37 (28.5–49)
<40 years	153 (58.2%)
>40 years	110 (41.8%)
Sex	
Male	166 (63.1%)
Female	97 (36.9%)
Presenting symptom	
Bleeding	154 (58.6%)
Obstruction	49 (18.6%)
Altered bowel habits	34 (12.9%)
Others	22 (8.4%)
Anemia	4 (1.5%)
Primary site	
Rectum	193 (73.4%)
Colon	70 (26.6%)
Clinical stage	
I-II	18 (6.8%)
III	162 (61.6%)
IV	83 (31.6%)
Differentiation	
Pure signet ring	183 (69.6%)
Mixed (signet + Mucinous)	80 (30.4%)
Pre-treatment CEA, ng/mL	
<5 ng/mL	122 (46.4%)
>5 ng/mL	140 (53.2%)
MSI testing	
MSI-high	19 (15.7%)
MSS	36 (29.8%)
MSI-low/pMMR	15 (12.4%)
Not done	51 (42.1%)

The rectum was the predominant primary tumour site, accounting for 193 (73.4%) cases, whereas only 70 (26.6%) originated in the colon, highlighting the marked rectal predominance. Most patients presented with advanced disease, with stage III constituting 162 (61.6%) cases and stage IV accounting for 83 (31.6%), while early-stage disease (stages I-II) represented only 18 (6.8%) of the cohort. Histologically, pure signet-ring cell carcinoma was observed in 183 (69.6%) patients, whereas 80 (30.4%) had mixed tumours containing both signet-ring and mucinous components. More than half of the patients, 140 (53.4%), had elevated CEA levels (≥5 ng/mL), suggesting a high tumour burden in a substantial proportion of patients. Microsatellite instability (MSI) testing was performed in only a subset of patients, among whom MSI-high tumours accounted for 19 (15.7%). Testing was unavailable in 42.1% of patients.

Nearly three-quarters of the patients, 194 (73.8%), underwent treatment with curative intent, while 69 (26.2%) received palliative therapy (Table 2). TNT followed by surgery was the most common treatment approach, 71 (27.2%), followed by upfront surgery, 58 (22.2%), neoadjuvant chemoradiotherapy followed by surgery, 50 (19.2%), and neoadjuvant chemotherapy alone followed by surgery, 15 (5.7%). Among the cohort of 12 patients who achieved complete response (CR), the majority underwent TNT followed by surgery (58.3%), while the remaining 41.7% received neoadjuvant chemoradiotherapy (NACT-RT) followed by surgery. Among the 64 patients with partial response (PR), TNT followed by surgery was also the most common treatment sequence (54.7%), followed by NACT-RT followed by surgery (35.9%) (Table 3). Disease progression despite neoadjuvant treatment occurred in 25 (22.9%), emphasising the aggressive biological behaviour of this tumour subtype. Surgical management was predominantly performed through an open approach in 82 (61.7%) patients, with laparoscopic surgery in 44 (33.1%) and robotic surgery in seven (5.3%) being less common. Adjuvant chemotherapy, either CAPOX or FOLFOX, was administered to most eligible patients, 104 (84.6%).

**Table 2:** Treatment received by the patients NACT: neoadjuvant chemotherapy, NACT-RT: neoadjuvant chemoradiotherapy, TNT: total neoadjuvant therapy.

Characteristics	Value
Treatment intent	
Curative	194 (73.8%)
Palliative	69 (26.2%)
Treatment sequence	
Upfront surgery	58 (22.2%)
NACT followed by surgery	15 (5.7%)
NACT-RT followed by surgery	50 (19.2%)
Total neoadjuvant therapy (TNT) followed by surgery	71 (27.2%)
Palliative chemotherapy	67 (25.7%)
Post-treatment response (clinical/radiological)	
Complete	12 (11%)
Partial	64 (58.7%)
No response/stable disease	8 (7.3%)
Progression	25 (22.9%)
Surgery	
Open	82 (61.7%)
Laparoscopic	44 (33.1%)
Robotic	7 (5.3%)
Adjuvant chemotherapy	
Received	104 (84.6%)
Not received	19 (15.4%)
Adjuvant radiotherapy	
Received	3 (6.8%)
Not received	41 (93.2%)

**Table 3:** Post-NACT response of the patients based on the treatment sequence NACT: neoadjuvant chemotherapy, NACT-RT: neoadjuvant chemoradiotherapy, TNT: total Neoadjuvant therapy, CR: complete response, PR: partial response, SD: stable disease, PD: progressive disease.

Treatment Sequence	Post NACT Response			
	CR (n = 12)	PR (n = 64)	No response (SD) (n = 8)	PD (n = 25)
NACT followed by surgery	0 (0%)	5 (7.8%)	1 (12.5%)	6 (24%)
NACT-RT followed by surgery	5 (41.7%)	23 (35.9%)	0 (0%)	4 (16%)
TNT followed by surgery	7 (58.3%)	35 (54.7%)	7 (87.5%)	14 (56%)

The median number of lymph nodes examined was 18 (IQR: 12.8-28) (Table 4), indicating adequate nodal assessment according to oncological standards. Among 119 patients who underwent curative surgical resection with available pathological lymph node assessment, the median number of positive lymph nodes was 2 (IQR: 0-7), and lymph node metastasis was identified in 74 patients (62.2%). Lymphovascular invasion (LVI), 28 (23.9%), and perineural invasion (PNI), 25 (21.4%), were observed in approximately one-fifth of patients, both representing adverse pathological prognostic features. Surgical resection margins were negative in 113 (93.4%) patients, reflecting a high rate of complete oncological resection. Nearly half, 54 (48.6%), developed recurrent disease during follow-up. The peritoneum was the most common site of recurrence, 20 (36.4%), followed by local recurrence, 11 (20.0%), distant metastasis, 10 (18.2%), nodal recurrence, 9 (16.4%), and multiple-site recurrence, 5 (9.1%). 

**Table 4:** Summarization of patient’s pathological characteristics underwent surgery. LVI: lymphovascular invasion, PNI: perineural invasion.

Characteristics	Value
Total lymph nodes examined	
Median (IQR)	18 (12.8-28)
Node positivity	
Positive (≥1 positive node)	74 (62.2%)
Negative (0 positive nodes)	45 (37.8%)
Pathological T stage	
pT0	23 (18.7%)
pT1-pT2	14 (11.4%)
pT3-pT4	86 (70%)
Pathological N stage	
pN0	45 (37.2%)
pN1	32 (26.4%)
pN2	44 (36.4%)
LVI	
Present	28 (23.9%)
Absent	89 (76.1%)
PNI	
Present	25 (21.4%)
Absent	92 (78.6%)
Resection margin	
Positive	8 (6.6%)
Negative	113 (93.4%)
Recurrence	
Present	54 (48.6%)
Absent	57 (51.4%)
Site of recurrence	
Peritoneum	20 (36.4%)
Local	11 (20.0%)
Distant	10 (18.2%)
Nodal	9 (16.4%)
Multiple sites	5 (9.1%)

**Figure 1:**
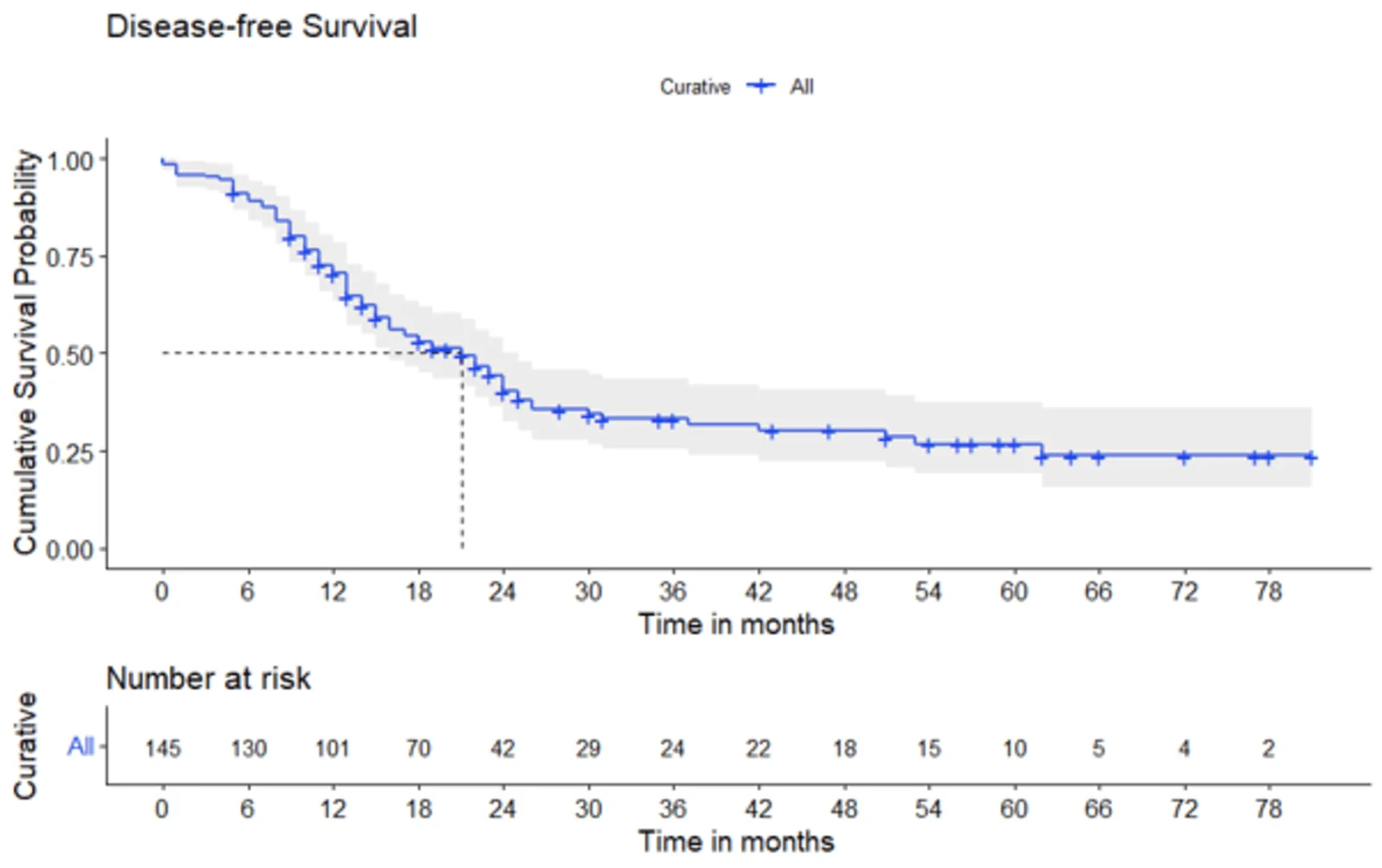
Kaplan–Meier curve for disease-free survival among patients treated with curative intent.

**Figure 2:**
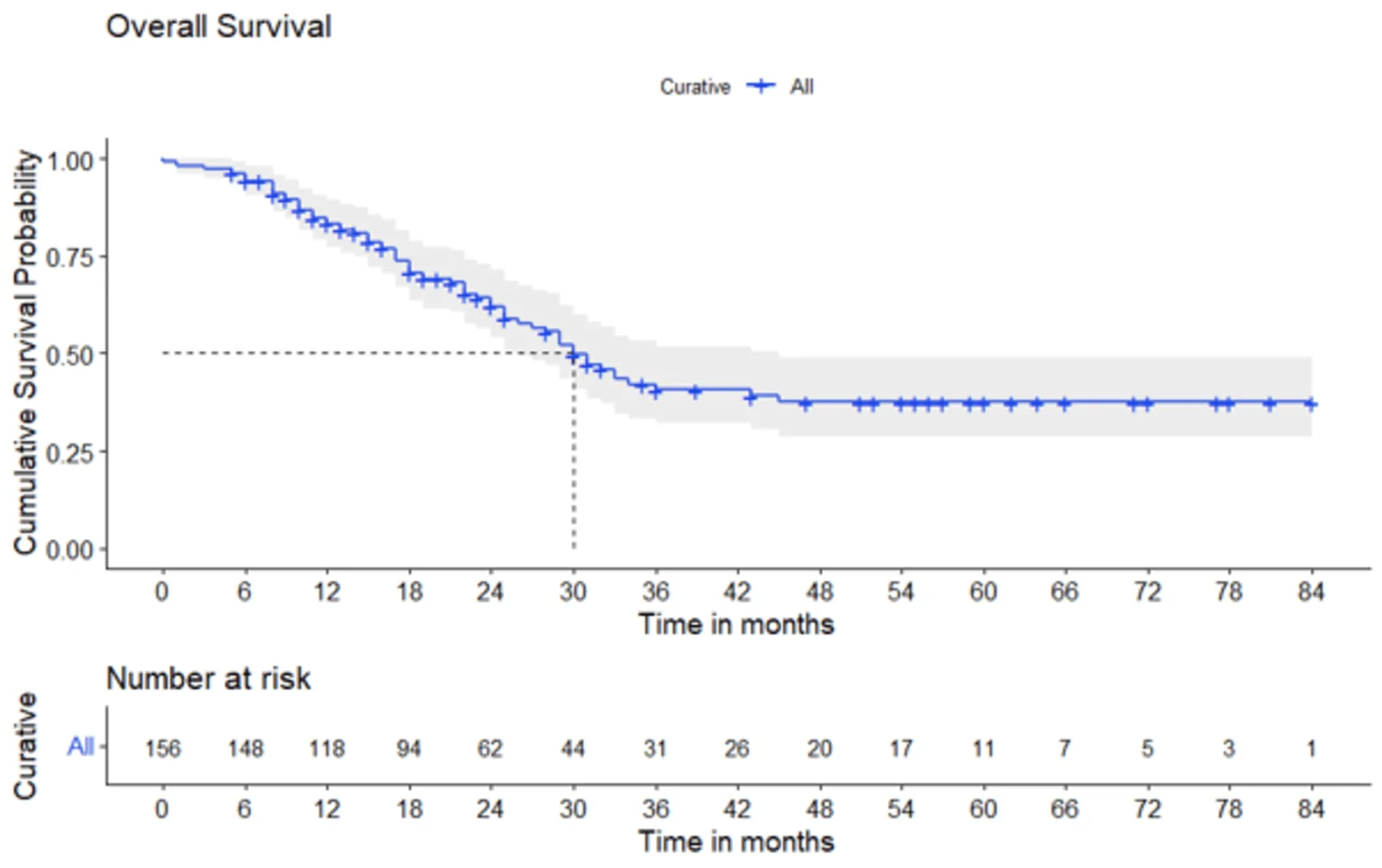
Kaplan–Meier curve for overall survival among patients treated with curative intent.

The median follow-up duration of the cohort was 35 months (IQR: 19-56). During follow-up, DFS was poorer, with a median DFS of only 21 months (95% CI: 15.8-26.1) (Figure 1). The estimated two-year DFS rate was 40.3 ± 4.5% (Figure 1), whereas the median overall survival was 30 months (95% CI: 26.1-33.9), reflecting the aggressive clinical course of the disease. The estimated two-year overall survival rate was 62.1 ± 4.4%, indicating that more than half of the patients survived beyond two years (Figure 2). 

On univariate analysis (Table 5), positive lymph node status, LVI, PNI, positive resection margins, and elevated baseline CEA were each significantly associated with worse overall survival (OS), whereas receipt of neoadjuvant treatment was associated with improved OS. On multivariable analysis, pathological nodal (pN) stage, resection margin status, and elevated baseline CEA retained borderline associations with worse OS after adjustment; however, none reached statistical significance in the reduced-sample model.

**Table 5:** Univariate and multivariate cox PH regression analysis to identify factors influencing overall survival of the patients. CEA: carcinoembryonic antigen; LVI: lymphovascular invasion; pN stage: pathological N stage; PNI: perineural invasion; pT stage: pathological T stage.

Factors	Univariate			Multivariate		
	HR	95% CI	p	HR	95% CI	p
Age	0.92	0.67–1.26	0.60	-	-	-
Sex	0.77	0.56–1.06	0.11	-	-	-
Elevated CEA	2.43	1.74–3.38	<0.001	1.98	0.94–4.18	0.07
Primary site	1.39	0.96–2.00	0.08	1.44	0.52–4.02	0.48
pT stage	1.06	0.41–2.69	0.91	-	-	-
pN stage	3.57	1.65–7.76	0.001	2.20	0.89–5.40	0.09
LVI	1.91	1.02–3.60	0.04	1.10	0.47–2.56	0.83
PNI	2.12	1.11–4.03	0.02	1.60	0.65–3.98	0.31
Margin status	2.65	1.11–6.35	0.03	2.51	0.95–6.65	0.06
Neoadjuvant treatment	0.49	0.34–0.69	<0.001	0.62	0.23–1.66	0.34
Adjuvant chemotherapy	0.69	0.31–1.56	0.37	-	-	-

## Discussion

SRCC of the colorectum is a rare but highly aggressive histological subtype characterised by distinctive clinicopathological and molecular features that differentiate it from conventional AC [8]. One of the most striking findings of the present study was the remarkably young age at diagnosis. The median age of our cohort was only 37 years, with nearly 153 (60%) patients being younger than 40 years. This is substantially younger than that reported in large Western population-based studies, including SEER analyses, where the median age at diagnosis generally ranges from 55 to 65 years despite SRCC occurring at a younger age than conventional AC [2,9-12]. Likewise, although the recent Indian study by Kazi et al. highlighted the disproportionately high prevalence of colorectal SRCC in the Indian subcontinent, the age at presentation in our cohort was even younger than that reported in their series [3,13], suggesting that our population represents an exceptionally early-onset subgroup. The reasons underlying this younger age distribution may include genetic susceptibility, environmental and dietary influences, referral bias to a tertiary cancer centre, or the increasing global incidence of early-onset colorectal cancer. Of 263 patients, only six (2.3%) were younger than 18 years. Given this very small number, a separate subgroup analysis comparing paediatric and adult patients would have been statistically underpowered and unlikely to yield reliable or clinically meaningful conclusions. Colorectal SRCC has generally been reported to be less likely to produce markedly elevated CEA levels than conventional AC. However, we found that 53.4% of patients had elevated baseline CEA (≥5 ng/mL). This may be explained by the fact that more than 90% of our cohort presented with stage III or IV disease, representing a high tumour burden at diagnosis. Elevated CEA in this setting may therefore reflect advanced disease rather than the intrinsic secretory characteristics of SRCC. In addition, we observed a male predominance (male-to-female ratio 1.7:1), which is consistent with most contemporary studies and systematic reviews reporting either a modest male predominance or a nearly equal sex distribution among patients with colorectal SRCC [9,14-16].

Unlike most Western population-based series, which have reported a predominance of proximal colonic tumours in colorectal SRCC, our cohort demonstrated a marked predominance of rectal cancers, accounting for 73.4% of all cases [8,17,18]. In contrast, studies from Asian countries have reported greater heterogeneity in tumour location [2,9]. This distribution is likely attributable to the tertiary referral nature of our institution rather than a true epidemiological difference. In India, many patients with colon cancer are treated at secondary or community hospitals, whereas those with locally advanced, recurrent, or technically challenging rectal cancers are more frequently referred to specialised colorectal and surgical oncology centres. Consequently, our cohort may overrepresent rectal cancers and younger patients with complex disease, limiting the external validity and generalisability of our findings to the broader population of patients with colorectal SRCC. Nevertheless, the study provides valuable real-world data on the management and outcomes of this rare malignancy in a high-volume tertiary cancer centre.

Consistent with previous reports, most patients in our study presented with advanced disease, with more than 90% diagnosed at stage III or IV. The aggressive behaviour of colorectal SRCC is largely attributed to alterations in cell adhesion pathways. Reduced or absent expression of E-cadherin, a calcium-dependent cell adhesion molecule, disrupts the E-cadherin/β-catenin complex, leading to loss of intercellular cohesion, diffuse tumour infiltration, and increased local invasiveness. Similar findings have been reported in gastric SRCC [19], where E-cadherin loss is associated with peritoneal dissemination and poor prognosis. Furthermore, abundant mucin production and overexpression of trefoil factors may further destabilise cell adhesion and facilitate tumour spread, while mucin may also impair immune recognition of tumour cells [20-25]. Collectively, these molecular alterations likely contribute to the advanced stage at presentation, frequent peritoneal metastasis, and poor prognosis of colorectal SRCC.

The poor survival observed in our cohort further highlights the aggressive nature of colorectal SRCC. The median DFS was only 21 months, with a two-year DFS rate of 40.3%, while the median overall survival was 30 months, with a two-year overall survival rate of 62.1%. These findings are consistent with previous studies [2,11,12] demonstrating inferior survival outcomes in SRCC compared with conventional AC, largely owing to advanced stage at presentation, frequent peritoneal dissemination, and aggressive tumour biology. Nearly half of the patients developed recurrence during follow-up, with the peritoneum being the most common site, consistent with the characteristic metastatic pattern reported in the literature [7,10,12].

Our survival analysis identified several pathological features associated with adverse prognosis in colorectal SRCC. On univariate analysis, positive lymph node status, LVI, PNI, positive resection margins, and elevated baseline CEA were significantly associated with worse overall survival, whereas receipt of neoadjuvant therapy was associated with improved survival. Elevated pretreatment CEA likely reflects greater tumour burden and advanced disease, explaining its association with inferior survival. Although pN stage, resection margin status, and elevated baseline CEA showed a strong trend towards worse overall survival, they did not reach statistical significance after adjustment in multivariable analysis. This is likely attributable to the limited number of events available for multivariable modelling, resulting in reduced statistical power, rather than the absence of a true biological association. Similar observations have been reported in previous studies of colorectal SRCC [2,8,12,15], where advanced nodal disease and incomplete resection consistently emerged as important determinants of survival.

This study has several limitations. First, its retrospective, single-centre design introduces the potential for selection and referral bias, which may limit the generalisability of our findings. Second, comprehensive molecular profiling, including MSI/MMR status and other biomarkers such as KRAS, NRAS, and BRAF mutation testing, was performed selectively based on institutional practice, clinical indications, and financial considerations rather than universally for all patients, limiting correlation between molecular characteristics and clinical outcomes. Third, the absence of a matched conventional AC control group precluded direct comparison of clinicopathological features and survival outcomes. Finally, although multivariable analyses identified trends towards poorer survival with nodal metastasis, positive resection margins, and elevated baseline CEA, the reduced number of events in the multivariable model may have limited statistical power to detect independent prognostic significance. Prospective multicentre studies with standardised treatment protocols and comprehensive molecular profiling are needed to validate these findings.

## Conclusions

Colorectal signet-ring cell carcinoma is an uncommon but highly aggressive malignancy characterised by early age at presentation, advanced disease stage, and poor oncological outcomes. Our findings highlight the unique demographic and clinicopathological profile of Indian patients with colorectal SRCC and reinforce the importance of early recognition and multidisciplinary management. Future collaborative prospective studies integrating molecular characterisation are warranted to improve risk stratification, optimise treatment selection, and ultimately enhance survival in this challenging disease.
